# Ligand-controlled diastereodivergent, enantio- and regioselective copper-catalyzed hydroxyalkylboration of 1,3-dienes with ketones†
†Electronic supplementary information (ESI) available. See DOI: 10.1039/c9sc03531a


**DOI:** 10.1039/c9sc03531a

**Published:** 2019-08-20

**Authors:** Jian-Jun Feng, Yan Xu, Martin Oestreich

**Affiliations:** a Institut für Chemie , Technische Universität Berlin , Strasse des 17. Juni 115 , 10623 Berlin , Germany . Email: martin.oestreich@tu-berlin.de

## Abstract

A copper-catalyzed three-component coupling of 1,3-dienes, bis(pinacolato)diboron, and ketones allows for the chemo-, regio-, diastereo- and enantioselective assembly of densely functionalized tertiary homoallylic alcohols. The relative configuration of the vicinal stereocenters is controlled by the chiral ligand employed. Subsequent transformations illustrate the versatility of these valuable chiral building blocks.

## Abstract 2

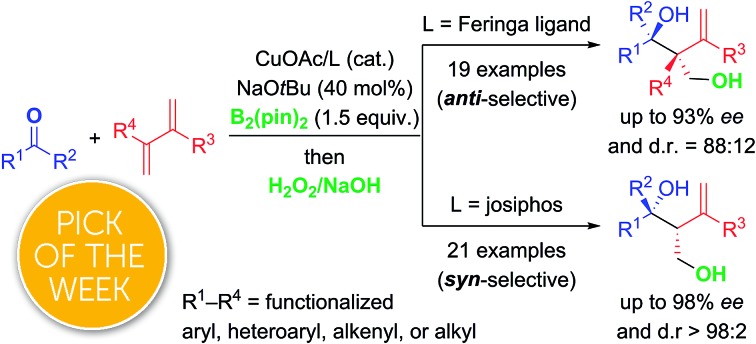
A ligand-controlled diastereodivergent copper-catalyzed borylative coupling between 1,3-dienes and ketones enables the enantioselective synthesis of densely functionalized tertiary homoallylic alcohols.

## Introduction

**Scheme 1:**
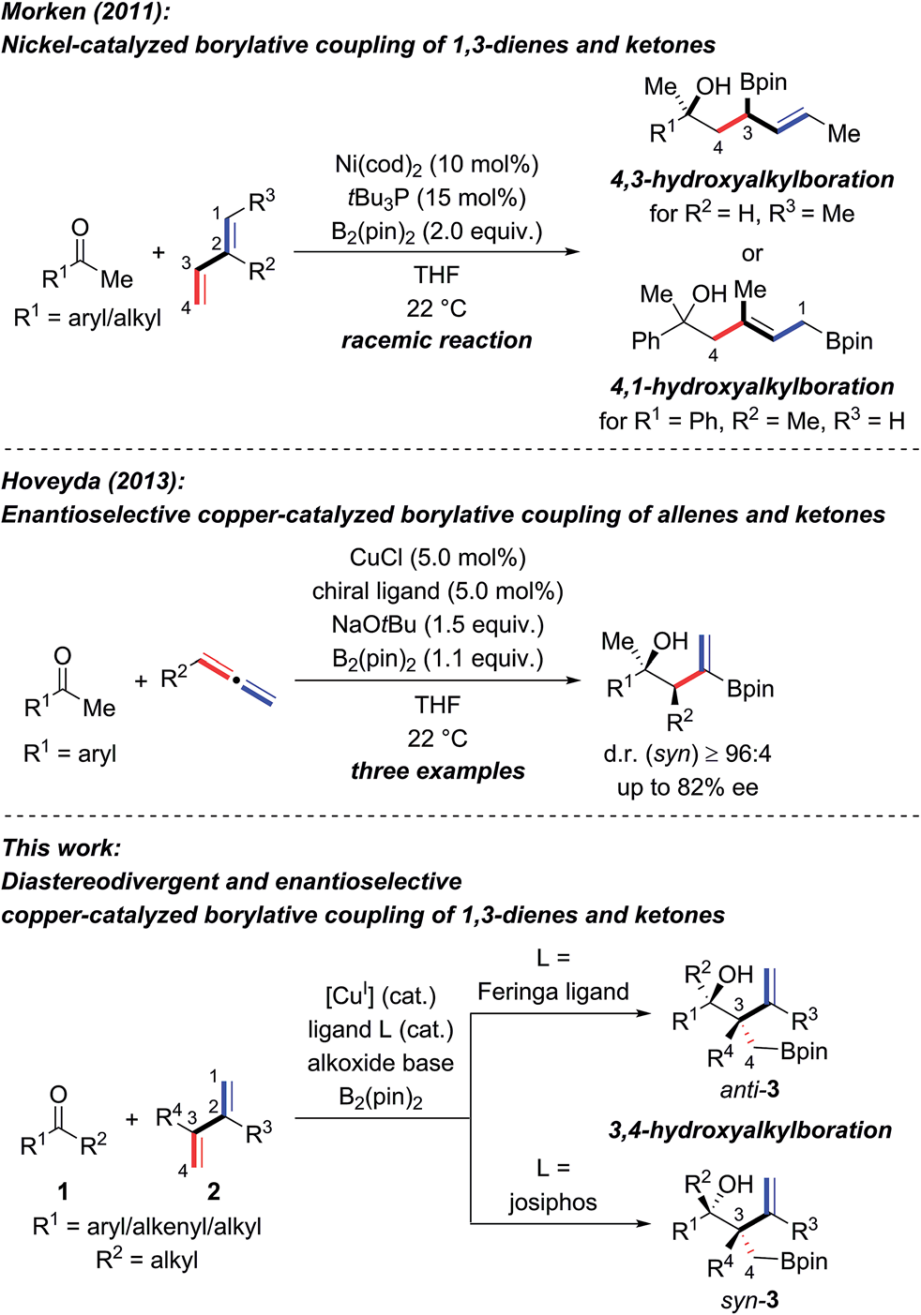
Transition-metal-catalyzed intermolecular borylative coupling reactions of ketones for the construction of tertiary homoallylic alcohols. cod = cycloocta-1,5-diene, pin = pinacolato.

The enantioselective synthesis of tertiary homoallylic alcohols^1^ continues to attract attention as these are highly useful intermediates in complex molecule synthesis and for medicinal chemistry.^2^ An established way to access that motif is by ketone allylation^3^–^7^ where enantiofacial discrimination and low reactivity are the key challenges compared to aldehydes as electrophiles.^8^ Many methods are based on preformed allylmetal reagents.^3^–^6^ An alternative to these nucleophiles is their *in situ* formation by hydrometalation of 1,3-dienes^9^,^10^ and allenes,^10^ and examples of transition-metal-catalyzed reductive couplings with ketones were recently achieved.^10^–^12^ A powerful variation of this approach is the borylmetalation of 1,3-dienes in the presence of a carbon electrophile.^13^–^17^ These and related stereoselective borylative coupling reactions of other π-systems form a carbon–boron and a carbon–carbon bond in a single operation.^13^ However, reactions involving ketones as electrophiles are scarce.^14^,17a,d–g To the best of our knowledge, there are only three examples of the preparation of tertiary homoallylic alcohols by the borylative coupling strategy. Morken and co-workers reported a nickel-catalyzed three-component coupling of 1,3-dienes, bis(pinacolato)diboron, and ketones in racemic fashion (Scheme 1, top).^14^ The reaction outcome was dependent on the substitution pattern of the 1,3-diene; (*E*)-penta-1,3-diene converted into 4,3-hydroxyalkylboration products while isoprene (one example) afforded the 4,1-hydroxyalkylboration product. Starting from allenes as the precursor of the allylic nucleophiles, Hoveyda and co-workers realized enantioselective borylative couplings with carbonyl compounds with *syn* selectivity but enantiocontrol was lower for ketones than for aldehydes (Scheme 1, middle).17*a* Low enantioselectivity was found by Tian and Tao in an intramolecular borylative cyclization of allenes tethered to cyclohexanediones (not shown).17*f* Hence, there is a demand for the development of new enantioselective borylative coupling reactions of π-systems and ketones to access chiral tertiary homoallylic alcohols. We disclose here such a copper-catalyzed three-component reaction with 1,3-dienes as the allylic coupling partner where the diastereoselectivity is determined by the ligand (Scheme 1, bottom).9d,e

## Results and discussion

For optimization, the three-component reaction of acetophenone (**1a**), isoprene (**2a**), and B_2_(pin)_2_ was chosen as the model reaction. The ligand effects are summarized in Table 1. In general, the reaction catalyzed by CuCl and phosphoramidite ligands afforded *anti*-**4aa** as the major diastereomer after oxidative degradation of the carbon–boron bond (see the ESI† for the complete set of data).^18^ As an example, *anti*-**4aa** formed in decent yield and with moderate stereoselectivity at room temperature in the presence of CuCl/**L1** and NaO*t*Bu (entry 1). Further optimization of the copper source, solvent, and temperature led to a system which afforded the tertiary homoallylic alcohol *anti*-**4aa** as the major diastereomer in 94% NMR yield and with 90% ee (entries 2–4). In contrast to phosphoramidite ligands, bisphosphine ligands commonly used in copper catalysis such as **L2** to **L12** furnished *syn*-**4aa** as the major diastereomer at room temperature (entries 5–17), and commercially available josiphos derivative **L9** was found to be optimal (entry 12). Lowering of reaction temperature from room temperature to –20 °C increased the enantiomeric excess and diastereoselectivity significantly but was detrimental to the yield (entry 13). Finally, high yield (98% NMR yield) and stereoselectivity (93% ee and d.r. = 87 : 13 in favor of *syn*) were restored in toluene/THF 8 : 2 with 5.0 mol% CuOAc and 6.0 mol% **L9** as the catalyst–ligand combination (entry 14).

**Table 1:** Selected examples of the optimization of the borylative hydroxyalkylation of 1,3-dienes^*a*^

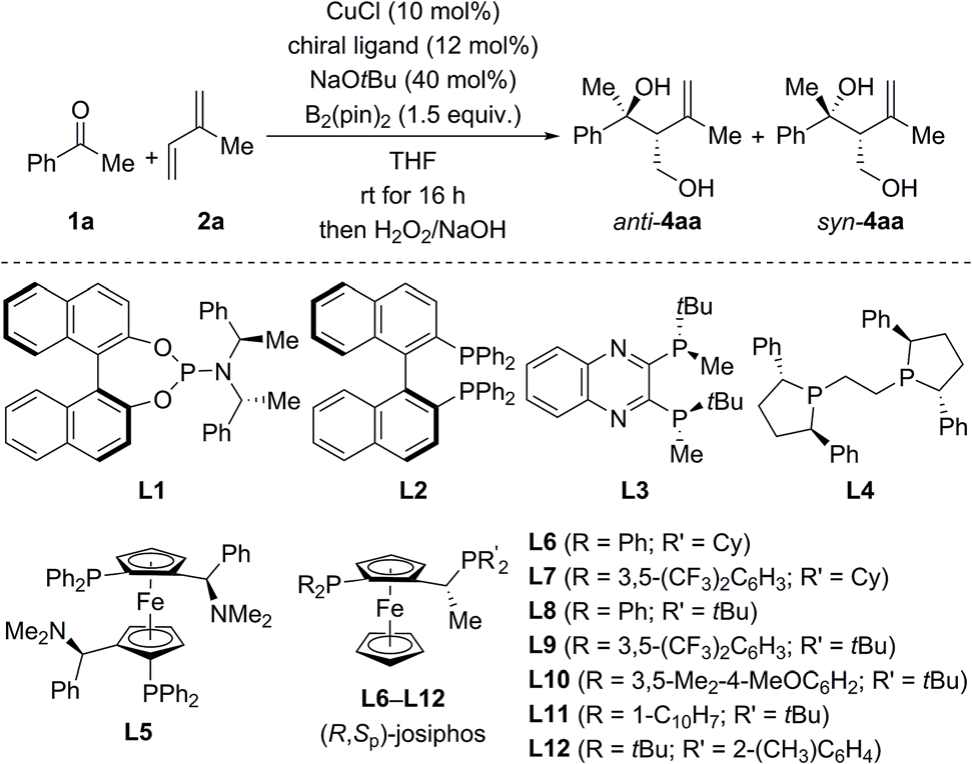					
Entry	Ligand	Yield^*b*^ (%)	d.r. (*anti* : *syn*)	ee^*c*^ (%)	
				*anti*-**4aa**	*syn*-**4aa**
1	**L1**	53	71 : 29	60	21
2^*d*^	**L1**	88	66 : 34	64	10
3^*d*^ ^,^^*e*^	**L1**	96	68 : 32	68	30
4^*d*^ ^,^^*e*^ ^,^^*f*^	**L1**	**94**	**80 : 20**	**90**	**64**
5	**L2**	75	42 : 58	43	32^*g*^
6	**L3**	92	35 : 65	6	35
7	**L4**	93	28 : 72	35^*g*^	32^*g*^
8^*e*^	**L5**	84	44 : 56	13	22
9	**L6**	45	23 : 77	22^*g*^	61
10	**L7**	98	23 : 77	13^*g*^	80
11	**L8**	80	22 : 78	72^*g*^	88
12	**L9**	98	23 : 77	74^*g*^	88
13^*h*^	**L9**	61	15 : 85	79^*g*^	94
14^*h*^ ^,^^*i*^	**L9**	**98**	**13 : 87**	**71** ^*g*^	**93**
15	**L10**	65	28 : 72	71^*g*^	87
16	**L11**	37	47 : 53	0	37
17	**L12**	29	49 : 51	—	—

^*a*^Unless otherwise noted, the reactions were performed with **1a** (0.2 mmol), **2a** (1 mmol), and B_2_(pin)_2_ (0.3 mmol) in THF (2 mL).

^*b*^Combined NMR yield determined by ^1^H NMR spectroscopy with CH_2_Br_2_ as an internal standard.

^*c*^Determined by HPLC analysis on chiral stationary phases.

^*d*^CuOAc instead of CuCl.

^*e*^Toluene instead of THF.

^*f*^Run at –30 °C.

^*g*^The other enantiomer was obtained.

^*h*^Run at –20 °C.

^*i*^0.4 mmol scale, 5.0 mol% CuOAc and 6.0 mol% **L9** were used and toluene/THF 8 : 2 instead of THF.

**Scheme 2:**
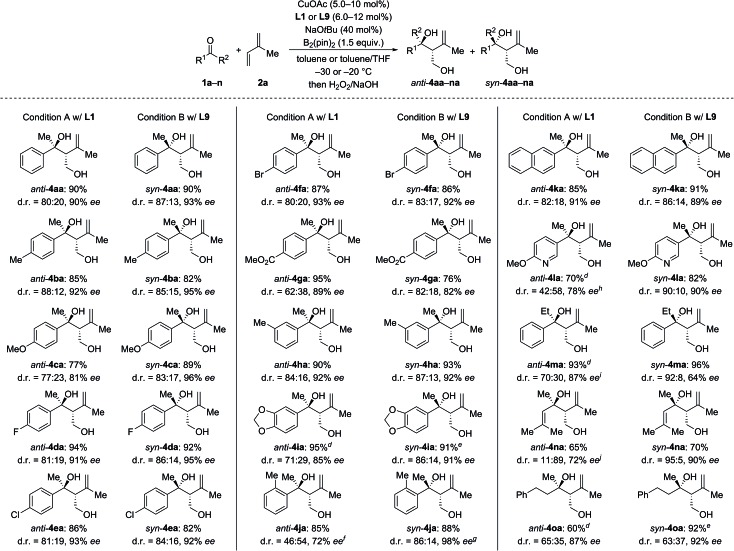
Scope I: variation the ketone.^a–c a^Condition A: CuOAc (10 mol%), **L1** (12 mol%), NaO*t*Bu (40 mol%), ketone **1** (0.20 mmol), isoprene (**2a**, 1.0 mmol), and B_2_(pin)_2_ (1.5 equiv.) in toluene (2 mL) at –30 °C. Condition B: CuOAc (5.0 mol%), **L9** (6.0 mol%), NaO*t*Bu (40 mol%), ketone **1** (0.40 mmol), isoprene (**2a**, 2.0 mmol), and B_2_(pin)_2_ (1.5 equiv.) in toluene/THF – 8 : 2 (3.5 mL) at –20 °C. ^b^Yields are combined isolated material; diastereomers are usually separable by flash chromatography on silica gel. ^c^The enantiomeric excess of the major diastereomer was determined by HPLC analysis on chiral stationary phases. ^d^CuOAc (15 mol%) and **L1** (18 mol%) were used. ^e^CuOAc (10 mol%) and **L9** (12 mol%) were used. ^f^*anti*-**4ja**: 29% ee. ^g^*anti*-**4ja**: 80% ee. ^h^ee value of *anti*-**4la**. ^i^*syn*-**4ma**: 78% ee. ^j^*syn*-**4na**: 72% ee.

We next investigated the scope of ketones using **L1** in the *anti*-selective procedure and **L9** in the *syn*-selective setup (Conditions A and B, Scheme 2). Acetophenones with various substituents in the *para* position, including electron-donating groups (as in **1b**, **c**) and halogens (as in **1d–f**), exhibited high reactivity and stereoselectivity. A carboxyl group was compatible (as in **1g**), thus further emphasizing the functional-group tolerance of this reaction. **1h** and **i** with *meta* substitution also gave satisfactory results. The reaction of *ortho*-methyl-substituted **1j** was successful under Condition B and yielded *syn*-**4ia** with 98% ee (*anti*-**4ja**: 80% ee); conversely, poor stereoselectivity was obtained under Condition A. Pyridyl-substituted **1l** reacted smoothly under Condition B and furnished *syn*-**4la** with good diastereoselectivity (d.r. = 90 : 10) and enantioselectivity (90% ee); in turn, the reaction of **1l** under Condition A produced *anti*-**4la** with a moderate ee value. Aside from aromatic methyl ketones, propiophenone (**1m**), which had not been compatible with Morken's^14^ and Hoveyda's17*a* catalytic system (*cf.*Scheme 1), also furnished *anti*-**4ma** in excellent yield and good enantioselectivity with moderate diastereoselectivity under Condition A; B afforded the target compound in a similar yield yet with a high diastereomeric ratio and a markedly diminished ee value. Interestingly, α,β-unsaturated ketone **1n** reacted regioselectively (1,2- over 1,4-addition) with good to excellent diastereoselectivity; *syn*-**4na** was the major product under both Condition A and B. Moreover, dialkyl ketone **1o** converted into the corresponding products *anti*- and *syn*-**4oa** under A and B but with low diastereoselectivity likely due to the little steric differentiation between the methyl and methylene groups attached to the carbonyl carbon atom.

**Scheme 3:**
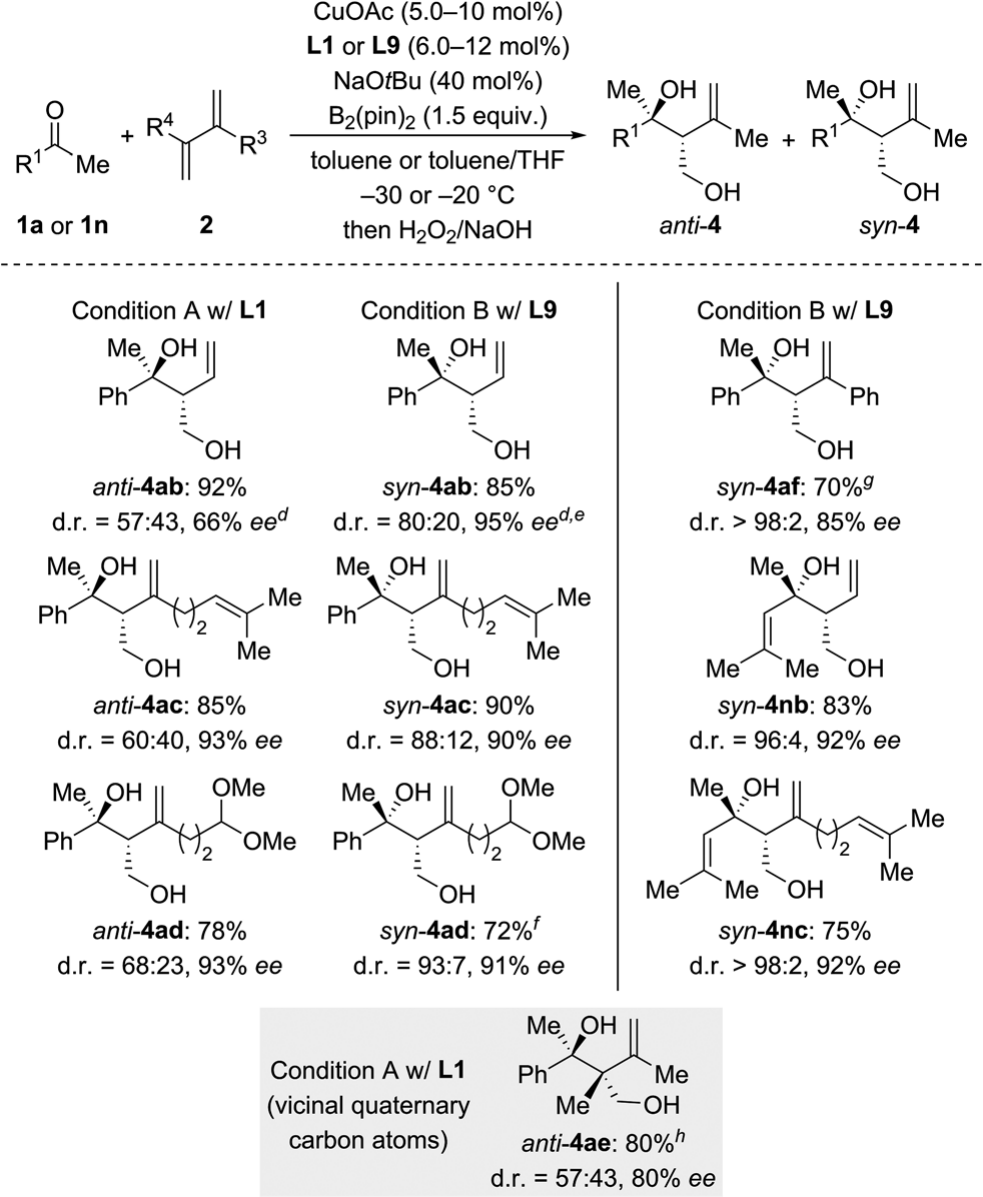
Scope II: variation of the 1,3-diene.^a–c^ For footnotes a–c, see Scheme 2. ^d^The absolute configuration was assigned by chemical correlation after separation of the diastereomers by flash chromatography on silica gel (see the ESI†). ^e^*anti*-**4ab**: 84% ee. ^f^CuOAc (8.0 mol%) and **L9** (10 mol%) were used. ^g^Run at –5 °C with CuOAc (10 mol%), **L9** (12 mol%), NaO*t*Bu (50 mol%), and B_2_(pin)_2_ (2.0 equiv.). ^h^CuOAc (15 mol%) and **L1** (18 mol%) were used.

We then examined the scope of 1,3-dienes (Scheme 3). Isoprene (**2a**) could be replaced by buta-1,3-diene (**2b**), myrcene (**2c**), its functionalized derivative **2d**, and 2,3-dimethylbuta-1,3-diene (**2e**). Yields were generally good but stereoselectivities ranged from poor to good under Condition A. In contrast, good to excellent stereoselectivities were observed for these 1,3-dienes under Condition B, *e.g.*, d.r. = 96 : 4 and 92% ee for **1n** → *syn*-**4nb** and d.r. = 93 : 7 and 91% ee for **1a** → *syn*-**4ad**. In the case of 2-aryl-substituted 1,3-diene **1f**, diastereodivergency was not achieved. Subjecting **1f** to Condition A afforded *syn*-**4af** in low yield as a single *syn*-isomer (not shown). However, applying Condition B at –5 °C significantly improved the yield and furnished the *syn*-**4af** with d.r. > 98 : 2 and 85% ee.

**Scheme 4:**
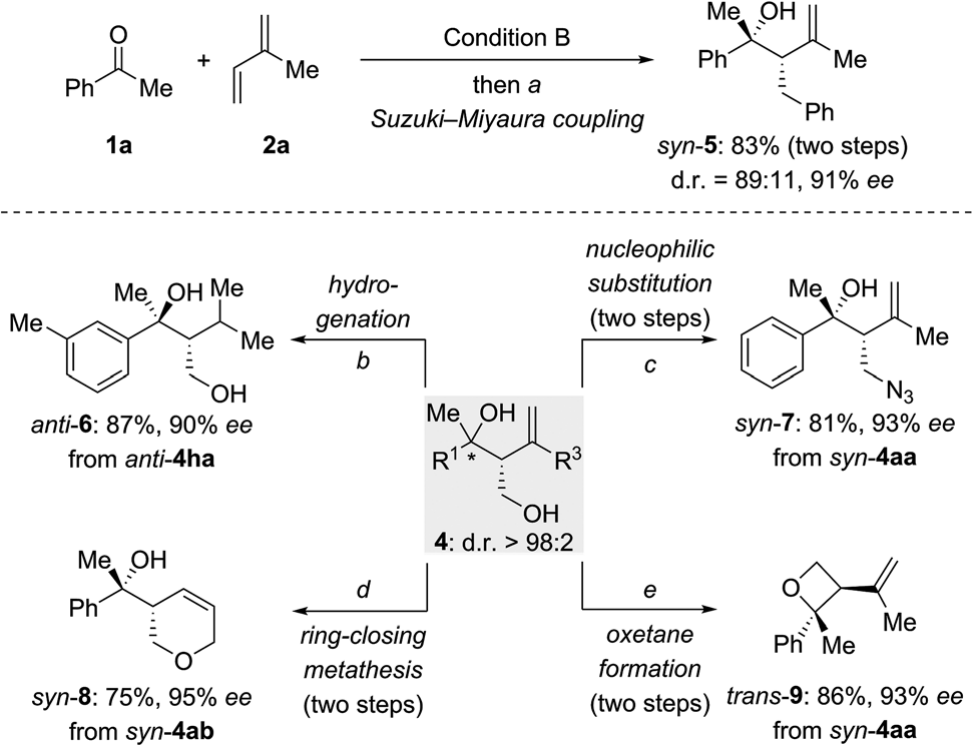
Tertiary homoallylic alcohols as versatile building blocks. (a) PhBr (1.8 equiv.), Pd(OAc)_2_ (5.0 mol%), RuPhos (10 mol%), KO*t*Bu (3.0 equiv.), toluene/H_2_O (10/1), 80 °C, 24 h; (b) Pd/C (10%), H_2_ (1 atm), MeOH, rt, 26 h; (c) (i) MsCl (1.5 equiv.), Et_3_N (1.5 equiv.), CH_2_Cl_2_, 0 °C to rt, 50 min; (ii) NaN_3_ (2.0 equiv.), DMF/H_2_O (10/1), 80 °C, 12 h; (d) (i) NaH (2.0 equiv.), allyl bromide (1.1 equiv.), THF, 0 °C to rt, 14 h; (ii) Hoveyda–Grubbs II (5.0 mol%), CH_2_Cl_2_, *Δ*, 12 h; (e) (i) TsCl (2.4 equiv.), pyridine, 0 °C to rt, 24 h; (ii) *n*BuLi (1.1 equiv.), –25 °C to rt, 15 h. Ms = methanesulfonyl.

To explore synthetic transformations of these tertiary homoallylic alcohols (Scheme 4), a scale-up synthesis of *syn*-**4aa** (1.0 mmol) under Condition B was done without any loss in efficiency and selectivity (see the ESI†). The primary alkyl borane generated by the multicomponent reaction was subjected to a Suzuki–Miyaura coupling to afford *syn*-**5** in 83% yield (Scheme 4, top). The versatility of the diol products **4** is illustrated for several transformations (Scheme 4, bottom). The 1,1-disubstituted double bond in *anti*-**4ha** was hydrogenated over Pd/C to produce *anti*-**6** in 87% yield. The hydroxy group in *syn*-**4aa** was replaced by an azide group through an S_N_2 reaction of an intermediate mesylate with NaN_3_ (*syn*-**4aa** → *syn*-**7**). Pyran *syn*-**8** was synthesized from *syn*-**4ab** by sequential alcohol allylation and ring-closing metathesis. Of note, a chemoselective tosylation of the primary alcohol in *syn*-**4aa** followed by a 4-*exo-tet* ring closure allowed for the construction of enantioenriched, trisubstituted oxetane *trans*-**9** in 86% yield.

## Conclusion

In summary, we have developed an efficient copper-catalyzed diastereodivergent and enantioselective borylative coupling of 1,3-dienes and ketones. Using a Feringa-type ligand **L1**, the reaction yielded *anti*-configured tertiary homoallylic alcohols while a switch to josiphos ligand **L9** resulted in *syn* selectivity (see the ESI† for a discussion of the reaction mechanism). This three-component coupling reaction represents a useful method for the preparation of stereochemically diverse tertiary alcohols bearing versatile alkenyl and boryl motifs from feedstock 1,3-dienes, ketones, and B_2_(pin)_2_. The synthetic utility of the reaction was showcased by several transformations.

## Conflicts of interest

There are no conflicts to declare.
